# Controlled synthesis and tunable properties of ultrathin silica nanotubes through spontaneous polycondensation on polyamine fibrils

**DOI:** 10.3762/bjnano.4.90

**Published:** 2013-11-25

**Authors:** Jian-Jun Yuan, Pei-Xin Zhu, Daisuke Noda, Ren-Hua Jin

**Affiliations:** 1 Synthetic Chemistry Lab., Kawamura Institute of Chemical Research, 631 Sakado, Sakura, 285-0078, Japan; 2 Department of Material and Life Chemistry, Kanagawa University, and JST-CREST 3-27-1 Rokkakubashi, Kanagawa-ku, Yokohama 221-8686, Japan

**Keywords:** biomimetic silicification, polyethyleneimine, silica–carbon nanocomposite, silica nanotubes, template synthesis

## Abstract

This paper describes a facile approach to a biomimetic rapid fabrication of ultrathin silica nanotubes with a highly uniform diameter of 10 nm and inner hollow of around 3 nm. The synthesis is carried out through a spontaneous polycondensation of alkoxysilane on polyamine crystalline fibrils that were conveniently produced from the neutralization of a solution of protonated linear polyethyleneimine (LPEI–H^+^) by alkali compounds. A simple mixing the fibrils with alkoxysilane in aqueous solution allowed for the rapid formation of silica to produce LPEI@silica hybrid nanotubes. These 10-nm nanotubes were hierarchically organized in a mat-like morphology with a typical size of 1–2 micrometers. The subsequent removal of organic LPEI via calcination resulted in silica nanotubes that keep this morphology. The morphology, the structure, the pore properties and the formation mechanism of the silica nanotubes were carefully investigated with scanning electron microscopy (SEM), transmission electron microscopy (TEM), Brunauer–Emmett–Teller measurements (BET), and X-ray diffraction (XRD). Detailed studies demonstrated that the formation of the nanotubes depends on the molar ratio of [OH]/[CH_2_CH_2_NH] during the neutralization as well as on the basicity of the alkali compound and on the concentration of the silica source. The synthesis of silica nanotubes established here could be easily applied to a fabrication on the kilogram scale. Silica nanotubes that were obtained from the calcination of hybrid nanotubes of LPEI@silica in an N_2_ atmosphere showed a distinct photoluminescence centered at 540 nm with a maximum excitation wavelength of 320 nm. Furthermore, LPEI@silica hybrid nanotubes were applied to create silica–carbon composite nanotubes by alternative adsorption of ionic polymers and subsequent carbonization.

## Introduction

Silica nanotubes with a controlled nanostructure (i.e., wall thickness and hollow space) and a tunable chemical composition are important for various applications, such as hydrogen storage [1], healthcare [2] and environmental technology [3]. It is well known that tubular silica structures can be fabricated by using inorganic [4], organic [5] or biological templates [6]. Among them, the utilization of self-assembled organic aggregates as templates for sol–gel reactions has received a great deal of attention, since this method is advantageous for controlling the structure, tuning the chemical composition and accessing surface functionalizations [7]. Shinkai and co-workers [8] reported the pioneering work on the formation of silica nanotubes by using a fibrous organogel as template for the silica deposition in mostly organic solvents. Also, silica nanotubes with helical character have been synthesized by employing chiral organogelating structures for the direct formation of silica [9]. Alternatively, self-assembled aggregates from surfactants or functional small molecules have also been developed for templating the formation of silica nanotubes [10–15]. However, these conventional methods cannot, in principle, avoid a non-templated silica deposition and also need harsh sol–gel conditions, such as long reaction times, elevated temperatures, and extreme pH values: This is partially due to the fact that the template aggregates cannot provide an efficient catalytic site for a selective silicification reaction. In addition, the organic template molecules normally need multiple steps to synthesize and thus are expensive, which limits a wider adoption and large-scale application of silica nanotubes [5,7–15].

In contrast, biosilicification in various biological systems such as diatoms and sponges proceeds in water under ambient conditions and produces siliceous skeletons with precisely controlled nanopatterns, a hierarchical morphology and organic–inorganic hybrid structures [16–18]. It has been demonstrated that the long-chain polyamines (partially combined with proteins) in diatom shells and the silicateins in marine sponges play a vital role in templating the biosilicification [18–19]. Therefore, a number of strategies to design self-assembled organic aggregates has been developed in order to make these organic matrices work as templates/scaffolds/catalysts for a deposition of silica at ambient conditions [20]. Recently, self-assembled fibrils of polypeptides [21–29] or amine-modified polysaccharides [30] have been used as template for the formation of silica nanotubes. For example, Yuwono et al. [23] reported the use of peptide-amphiphile nanofiber templates in order to direct the synthesis of hollow silica nanotubes with outer diameters of 15–23 nm. Pouget et al. [22] synthesized double-walled nanotubes that possess a silica/Lanreotide/silica wall architecture through a unique synergistic growth mechanism. With these methods, which are inspired by biosilicification, silica can be selectively deposited on the template under mild conditions. However, the formation of silica nanotubes needs relatively long reaction times (i.e., several days), and the use of the peptides as templates is costly. In order to achieve a large-scale commercial application of silica nanotubes, a highly efficient low-cost strategy based on simple synthetic chemistry is highly desirable. We are interested in the programmable construction of biomimetic silica nanomaterials by exploiting the crystallization-driven self-assembly of a simple synthetic polyamine, namely linear polyethyleneimine (LPEI) [31–33]. In contrast to branched PEI, LPEI is composed only of secondary amine (NHCH_2_CH_2_, EI unit) and is highly crystalline because of its linear structure [34]. We discovered that, when cooling its hot aqueous solution, LPEI tends to self-assemble into a crystalline nanofilament structure associated with water molecules ((NHCH_2_CH_2_)/2H_2_O) [35]. This crystalline nanofilament itself acts as a template that directs the silica morphology as well as a scaffold for the deposition of silica, and as a highly efficient catalyst for promoting the formation of silica. This leads to the facile formation of the one-dimensional LPEI@silica hybrid nanostructure. Further studies indicated that this method of cooling a hot solution normally produced a mixture of nanoribbons and nanofibers with diameters ranging from 30 to 150 nm [36]. A removal of the LPEI core from the nanofibers led to the formation of hollow silica but without control over the final structures. Currently, a facile fabrication of ultrathin (i.e., about 10 nm) high-quality silica nanotubes still remains a challenge.

In contrast to the free base LPEI, which is insoluble in water at room temperature, the protonated LPEI–H^+^ is freely soluble in water under ambient conditions. This feature is actually desirable for the self-assembly of LPEI from LPEI–H^+^ via a simple neutralization and deprotonation route at ambient temperature. Very recently, we have demonstrated the synthesis of thin films, which consisted of either LPEI@silica hybrid or silica nanotubes. This was achieved through a key step of the neutralization of LPEI–H^+^, which was absorbed at the substrate before [37]. That is, the substrate was dipped into an aqueous solution of LPEI–H^+^ to adsorb the polymers and then dipped into alkali solution for the neutralization. This self-assembly provided very thin films of LPEI fibrils that allowed for the controlled mineralization of silica, which resulted in hierarchically structured thin coatings composed of LPEI@silica hybrid nanotubes. However, the synthesis of silica nanotube powders through the alkali-induced self-assembly route was not studied systematically. In this paper, we examined the synthesis and the properties of the silica nanostructures in detail. The self-assembly of LPEI was promoted by adding alkali into aqueous solution of LPEI–H^+^. The transformation of the soluble protonated LPEI into the insoluble free base led to the growth of crystalline fibrils. These LPEI fibrils acted as template/scaffold/catalysts for the controlled silicification that afforded LPEI@silica hybrid nanotubes, which can be subsequently changed into pure silica nanotubes by removing the organic LPEI. Moreover, we also addressed the possibility to synthesize silica–carbon composite nanotubes by exploiting hybrid chemistry with LPEI@silica nanotubes. We confirmed that a room-temperature alkali-induced approach to the formation of silica yields highly controlled ultrathin silica nanotubes with uniform diameter of 10 nm and inner hollows of around 3 nm. The hybrid LPEI@silica nanotubes were further applied to create silica/carbon hybridized nanotubes by alternative adsorption of ionic polymers and subsequent carbonization.

## Results and Discussion

**Figure 1:**
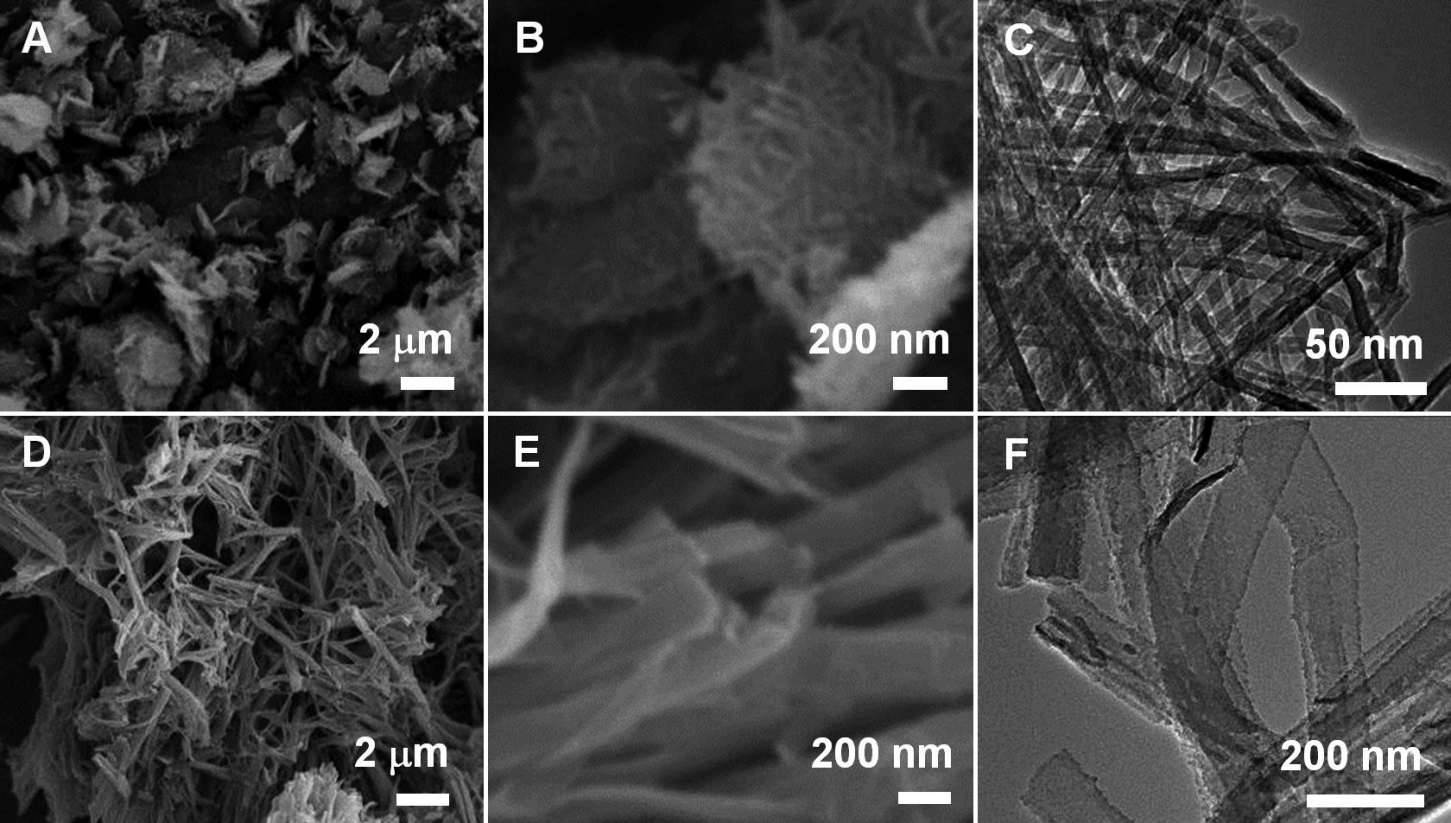
SEM (A, B, D and E) and TEM (C and F) images of silica nanotubes synthesized by alkali-induced room-temperature self-assembly of protonated LPEI (A–C) and silica nanoribbons formed by temperature-induced self-assembly of LPEI (D–F). The LPEI template for the formation of silica nanoribbons was prepared by naturally cooling a 10 mL of hot solution of 3 wt % LPEI. In order to synthesize the silica nanotubes, self-assembled LPEI aggregates were prepared by first dropping 1.0 mL of aqueous NaOH solution (5.0 M) into a mixture of 0.5 g of LPEI·HCl and 5 mL of water, and then washing the crystalline LPEI aggregates to pH 7.0 with centrifugation–redispersion cycles. The silica depositions are the same for the formation of nanotubes and nanoribbons. The latter was performed by adding 1.5 mL of MS51 into 15 mL of aqueous dispersion of LPEI aggregates for 1.0 h at room temperature.

**Silica nanotubes templated by alkali-induced LPEI fibrils.** Self-assembled LPEI fibrils were prepared by dropping NaOH solution (1 mL, 5 M) into 5 mL of an aqueous solution of LPEI·HCl (containing 0.5 g) with a molar ratio of [OH]/[EI] = 0.8 at room temperature. The resulting LPEI fibrils were dispersed in 15 mL of water (pH 7.2) and then mixed with 1.5 mL of methyl silicate 51 (MS51) and kept at room temperature for 1 h. The resulting LPEI@silica hybrid nanotubes as well as the products of a calcination at 800 °C were characterized by SEM and TEM. As shown in Figure 1A, two-dimensional silica mats with diameters of ca. 1–2 μm were observed. The high-magnification SEM image indicates that the mat is thin, and composed of very thin one-dimensional nanostructures (Figure 1B). To examine the structure of the nanotubes, the sample was further visualized by TEM. As shown in Figure 1C, the mat is densely knitted from straight one-dimensional nanotubes as the elemental nanostructure. The nanotubes are highly uniform with a diameter of 10 nm and a wall thickness of roughly 3 nm. While self-assembled polypeptides could template the formation of silica nanotubes with uniform diameter, it still remains challenge for the polypeptide template to synthesize stable silica nanotube structures with very small diameters (i.e., 10 nm) [21–27]. Moreover, polypeptides are unsuitable for the large-scale production of silica nanotubes. In contrast, LPEI is one of simplest synthetic polymers and its alkali-induced self-assembly can be easily performed in water at room temperature. According to the recipe to the silica nanotubes shown in Figure 1A–C, we succeeded in the kilogram scale synthesis. To verify the individual characteristic of the alkali-induced self-assembly, the silica formation templated by the LPEI aggregates obtained by cooling a hot LPEI solution procedure was carried out under comparative conditions and the product was subjected to SEM and TEM (see Figure 1D–F). Remarkably, a different silica structure of nanoribbons with a typical width of 100–200 nm was produced. This indicates that large ribbon-like aggregates of LPEI were induced when naturally cooling the hot solution of LPEI because of the relatively slow crystallization. In contrast, the alkali-induced self-assembly of LPEI occurred rapidly because the deprotonation reaction is a fast process. Comparatively, the LPEI fibrils from rapid crystallization-driven self-assembly serve as templates that allow for a well-controlled silicification.

**Figure 2:**
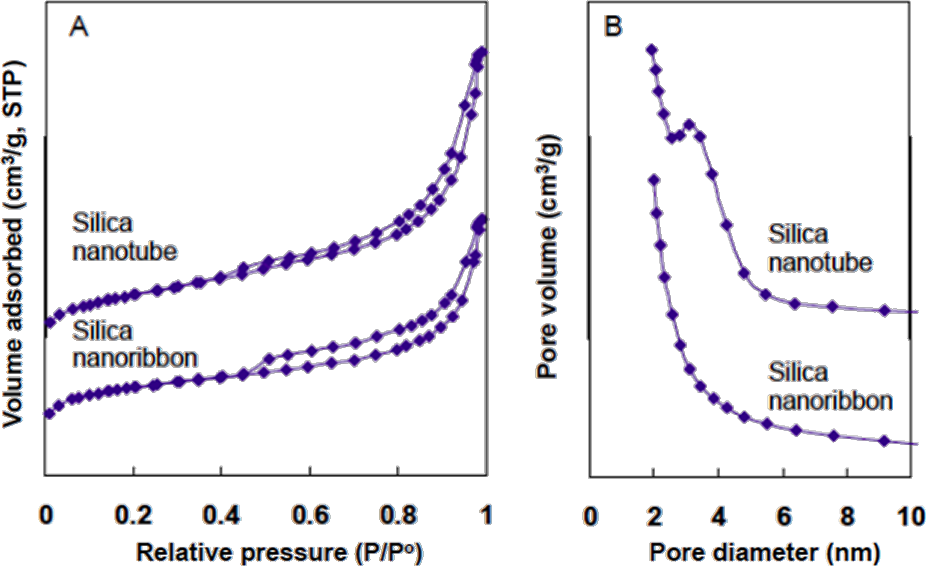
N_2_ adsorption/desorption isotherms (A) and BJH pore-size distribution curve obtained from the adsorption branch (B) of silica nanoribbon and silica nanotube. The synthesis conditions are the same to that of the samples shown in Figure 1. The sample was calcined at 800 °C in air for 3 h with at heating rate of 2.5 °C per min.

The surface areas and pore size distributions of the silica nanotubes and nanoribbons shown in Figure 1 were characterized with nitrogen adsorption and desorption measurements. As shown in Figure 2A, the BET adsorption–desorption isotherms of both silica nanotubes and nanoribbons can be described as type-IV hysteresis loops, which are indicative of the mesoporous nature of nanotubes and nanoribbons. The BET specific surface areas of the silica nanotubes and nanoribbons were calculated to be 307 m^2^/g (run 1 in Table 1) and 404 m^2^/g (run 2 in Table 1), respectively. Barrett–Joyner–Halenda (BJH) calculations derived from the adsorption branch showed that there is no peak value observed for silica nanoribbons. This suggests that the size of the mesopores arises from slit-like structures formed by randomly collapsed nanoribbons. In comparison, the silica nanotubes exhibited a narrow pore distribution with a peak value of around 3.5 nm, which corresponds to the hollow inner of nanotube (Figure 2B) [38–39], which is in good agreement with the TEM observations (Figure 1C). This BET result indicates that our silica nanotubes have an excellent thermal stability compared to the conventional mesoporous silica (i.e., M41S), which may collapse upon calcination at temperatures higher than 750 °C [40]. Regarding this, it should be noted that the conventional surfactant-based sol–gel reaction is catalyzed by HCl or NaOH. In this system, the silica sol forms in aqueous solution and then subsequently precipitates within the space of the template to form silica gel. In comparison, in our LPEI aggregate-based silica deposition, the hydrolysis and polycondensation of alkoxysilane occurs simultaneously and selectively on the surface of the LPEI templates. This characteristic hydrolytic polycondensation process gives a homogeneously structured SiO_2_ framework. We assume that such silica walls contribute to improve the thermal stability of silica nanotubes.

**Table 1:** Summary of the molar ratios of [OH]/[EI] for the synthesis of the LPEI templates, the TGA analysis of the composition of LPEI@silica hybrid nanostructures and BET data of the silica nanostructures obtained by calcination of LPEI@silica hybrid at 800 °C at air.^a^

run	ratio of [OH]/[EI] for self-assembly	TG analysis (weight loss %)	BET surface area (m^2^/g)	t-plot micropore area (m^2^/g)	t-plot external surface area (m^2^/g)	BJH desorption cumulative volume of pores (cm^3^/g)	maximum of pore width (nm)

1	0.8	30.6	356	61	295	0.673	3.9
2	0.8	24.6	320	82	238	0.558	4.2
3	0.8	23.5	317	91	226	0.533	4.2
4	0.56	19.0	146	33	114	0.395	—
5	3.2	28.68	360	25	335	0.698	3.53
6	0.8	18.9	244	114	130	0.279	—
7	0.8	25.8	387	81	306	0.696	4.2
8	0.8	25.3	294	60	234	0.538	4.3
9	0.8	25.3	419	86	333	0.740	4.2
10	0.8	26.64	477	251	226	0.45	—
11	3.2	29.6	430	162	268	0.625	—

^a^run 1: [NaOH]/[EI] = 0.8, silicification 5 min; run 2: [NaOH]/[EI] = 0.8, silicification 60 min; run 3: [NaOH]/[EI] = 0.8, silicification 240 min; run 4: [NaOH]/[EI] = 0.56, silicification 60 min; run 5: [OH]/[EI] = 3.2, silicification 60 min; run 6: [NaOH]/[EI] = 3.2, [MS-51] = 23.1 wt %, silicification 60 min; run 7: [NaOH]/[EI] = 0.56, [MS-51] = 9.1 wt %, silicification 60 min; run 8: [NaOH]/[EI] = 0.56, [MS-51] = 4.6 wt %, silicification 60 min; run 9: [NaOH]/[EI] = 0.8, [MS-51] = 1.1 wt %, silicification 60 min; run 10: [NH_4_OH]/[EI] = 0.8, MS-51/H_2_O = 1.5/15 (v/v), silicification 60 min; run 11: [NH_4_OH]/[EI] = 3.2, MS-51/H_2_O = 1.5/15 (v/v), silicification 60 min.

**Figure 3:**
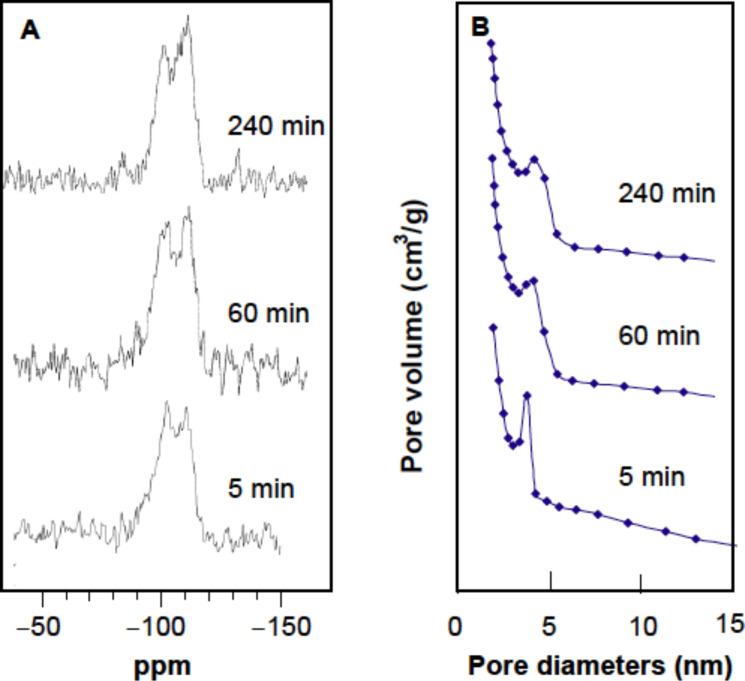
(A) Solid state ^29^Si CP MAS NMR spectra of LPEI@silica hybrid nanotubes formed after silicification times of 5 min, 60 min and 240 min. The LPEI aggregates were prepared by first dropping 1.0 mL aqueous NaOH solution (5.0 M) into a mixture of 0.5 g of LPEI·HCl and 5 mL of water ([OH]/[EI] = 0.8), then washing the crystalline LPEI aggregates to pH 7.0 with centrifugation–redispersion cycles. The silica deposition was performed by stirring a mixture of 1.5 mL of MS51 and 15 mL of aqueous dispersion of LPEI aggregates at room temperature. (B) BJH pore-size distribution curve obtained from the adsorption branch of the calcined nanotubes of the LPEI@silica hybrid nanotubes shown in (A).

**Silicification reaction time.** To evaluate the ability of LPEI fibrils to catalyze the formation of a silica framework, we performed solid-state ^29^Si CP-MAS NMR measurements of the LPEI@silica nanotubes, which were synthesized with different silicification times ranging from 5 to 240 min. The LPEI fibrils were prepared by dropping 1.0 mL of NaOH solution (5 M) into an aqueous solution containing 6.0 mL of water and 0.5 g of LPEI·HCl (molar ratio of [OH]/[EI] = 0.8) at room temperature. After water washing, the LPEI fibrils were dispersed into 15 mL of water with pH 7.2. To this dispersion, 1.5 mL of MS51 was added and the mixture was stirred at room temperature for different times. We found that the reaction at 5 min produced a silica framework with Q4/Q3 = 0.88 (no signal of Q2 detectable), indicating that silica with a high degree of polycondensation was produced within a very short time (Figure 3A). When the reaction times were increased up to 60 min and 240 min, the degree of polycondensation increased, which is shown by the ratios Q4/Q3 of 1.30 and 2.1, respectively. After removing the organic LPEI by calcining at 800 °C, the samples were examined by nitrogen adsorption and desorption measurements. It was surprising that only five minutes of the silicification reaction is enough to impart the nanosized hollow structure in the resulting silica, which was evidenced by BJH calculations from adsorption branch that shows a peak value of 3.9 nm corresponding to a nanotubular silica structure (Figure 3B and run 1 in Table 1). We also confirmed that this silica nanotube has the BET specific surface area of 356 m^2^/g (run 1 in Table 1 and Figure S1). The silica nanostructures from the hydrolytic condensation reaction times of 60 min and 240 min have the BET specific surface areas of 320 m^2^/g (run 2 in Table 1) and 317 m^2^/g, respectively (run 3 in Table 1). Their pore size distribution determined by the BJH calculations from adsorption branch has the same peak value of 4.2 nm (Figure 3B) indicating the existence of hollow structure. SEM images demonstrated that the nanotubes synthesized from 5 min, 60 min and 240 min have similar mat morphology (Figure S1 in Supporting Information File 1). Obviously, LPEI fibrils exhibited the high-efficient ability as template/scaffold/catalyst for rapid formation of silica-based wall with high toughness around polymer template via temporally and spatially controlled silicification. This rapid LPEI-mediated silicification would be complete when the reaction time increased longer than 60 min, due to that the catalytic LPEI has been significantly buried into silica matrix so that it became not available for further catalyzing the reaction.

**Figure 4:**
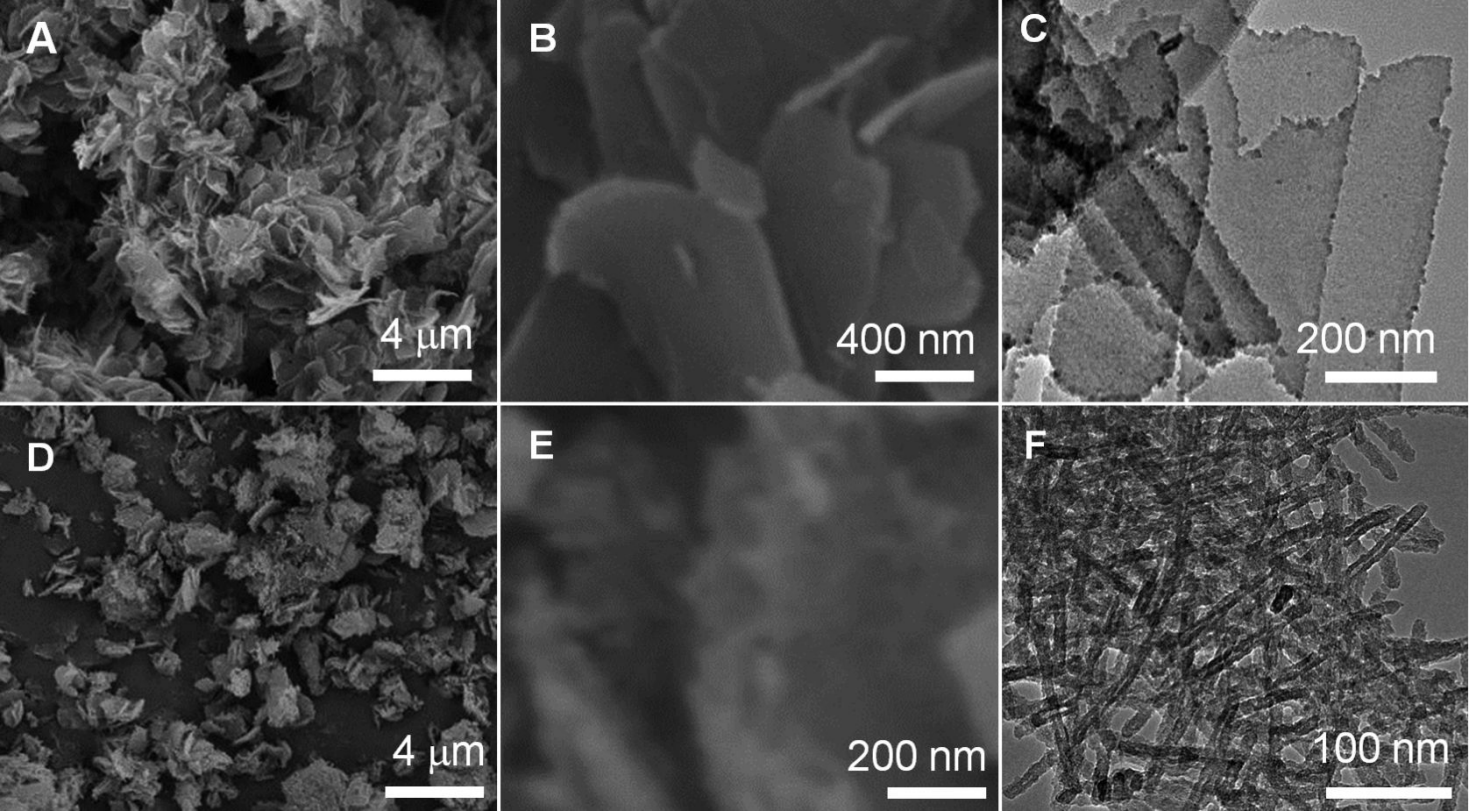
SEM (A, B, D and E) and TEM (C and F) images of silica prepared with molar ratios [OH]/[EI] of 0.56 (A–C) and 3.2 (D–F). The LPEI aggregates were formed first by dropping aqueous NaOH solution into a mixture of 0.5 g of PEI-HCl and 6 mL of water and then washing aggregates with water to pH 7.0. The silicification conditions are the same as described in Figure 1.

**Effect of the molar ratios of [OH]/[EI].** Furthermore, we addressed the dependence of the formation of silica nanotubes on the molar ratios of [OH]/[EI]. Firstly, we prepared self-assembled LPEI aggregates with a decreased molar ratio of [OH]/[EI]. For example, 0.7 mL of NaOH solution (5 M) was dropped into a solution containing 6.0 mL of water and 0.5 g of LPEI·HCl ([OH]/[EI] = 0.56). The mixture was stirred at room temperature for 24 h for LPEI self-assembly before washing and silicification. Silica deposition was performed by mixing 1.5 mL of MS51 and 15 mL of the aqueous dispersion of LPEI aggregates at room temperature for 1 h. As shown in Figure 4A and B, large aggregates composed of two-dimensional films were formed. TEM observation demonstrated that the nanofilm is highly transparent for TEM electron beam (Figure 4C), which suggests that the film is very thin. The BET studies indicated that the nanofilm has a relatively low surface area of 146 m^2^/g (run 4 in Table 1 and Figure S2 in Supporting Information File 1) and no peak value was observed in the pore distribution. Different to the silica nanotube formation from a higher molar ratio of [OH]/[EI] (0.8, Figure 1A–C), the decreased molar ratio of [OH]/[EI] induced the formation of nanofilms. This could be attributed to a slower crystallization rate of LPEI, because of insufficient neutralization of the protonated LPEI. On the other hand, when the molar ratio of [OH]/[EI] is higher than 0.8, the self-assembled LPEI fibrils could successfully template the formation of silica nanotubes. Figure 4D–F show typical electron microscopic images of silica nanotubes formed by using a molar ratio of [OH]/[EI] = 3.2. Both SEM (Figure 4D and E) and TEM (Figure 4F) images proved the formation of similar mat-like powders, which consist of silica nanotubes. BET studies showed a surface area of 360 m^2^/g and a BJH peak value of 3.5 nm for the tube structures (run 5 in Table 1 and Figure S2 in Supporting Information File 1). These values are similar to those achieved with a molar ratio [OH]/[EI] of 0.8.

**Figure 5:**
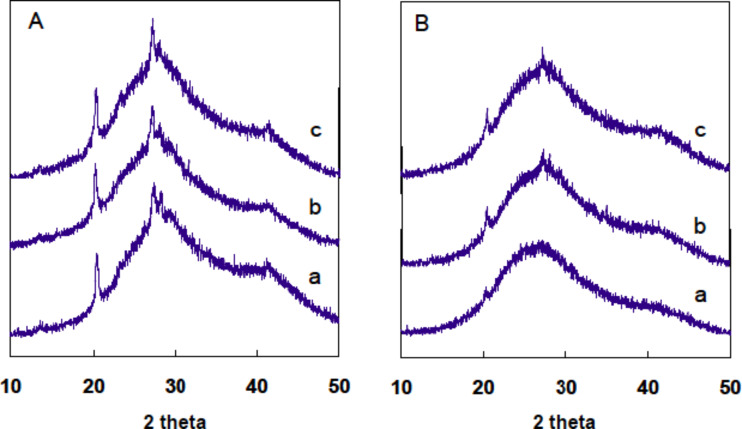
XRD profiles of LPEI aggregates (A) and LPEI@silica hybrid nanostructures (B). The LPEI aggregates were prepared by dropping 5.0 M NaOH solution into a mixture of 0.5 g of LPEI·HCl and 6 mL of water with ratios [OH]/[EI] of 0.8 (a), 1.6 (b) and 3.2 (c). The silica deposition conditions are the same for a, b and c: 1.5 mL of MS51 was added into 15 mL of aqueous dispersion of LPEI aggregates (pH 7.0) and stirred for 1 h at room temperature.

**Scheme 1:**
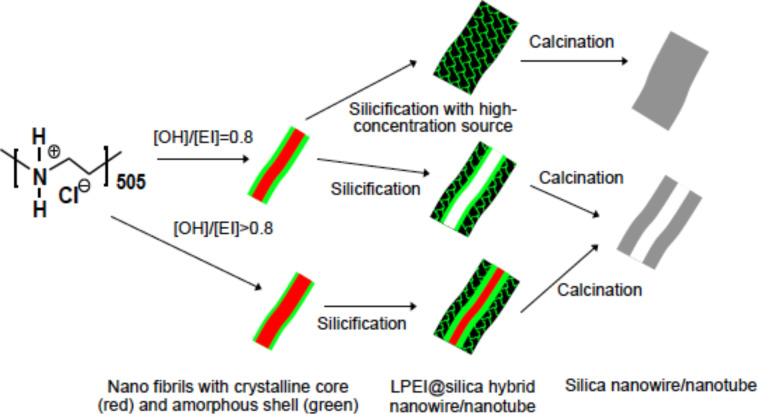
Proposed formation mechanism of the polyamine-aggregate template for silica nanotubes and nanowires formed by the alkali-induced self-assembly of LPEI.

XRD measurements were performed in order to investigate the template role of LPEI fibrils for the formation of nanotubes. Three types of LPEI fibrils were prepared with molar ratios of [OH]/[EI] at 0.8, 1.6 and 3.2. The silica depositions were performed with the same conditions as given in Figure 4. We found that the three LPEI samples showed strong diffraction peaks of the crystalline LPEI at 2θ = 23°, 27° and 30° (Figure 5A). After the deposition reaction on the template prepared from the molar ratio of [OH]/[EI] 0.8, the peaks at 2θ = 27° and 30° completely disappeared and the peak at 2θ = 23° became almost undetectable (Figure 5B): This indicated that the crystalline LPEI template has almost disappeared with the silica deposition and the formation of LPEI@silica hybrid tubular walls (Scheme 1). A similar phenomenon is seen well in our previous results, in which the silica deposition was carried out on the same LPEI template but with an extremely diluted silica source. In contrast, when using LPEI fibrils from the [OH]/[EI] molar ratios of 1.6 and 3.2 for silicification, the diffraction peaks due to crystalline LPEI template after silica deposition were obviously detected (Figure 5B). This means that the silicification only partially damaged the crystalline template. We speculate that the higher molar ratios [OH]/[EI] result in LPEI fibrils with an enhanced crystallization structure that can partially survive the silicification reaction. Therefore, it is reasonable to suggest that LPEI fibrils from higher molar ratios of [OH]/[EI] can allow for the formation of hybrid nanostructures that have a crystalline LPEI core and LPEI@silica hybrid wall (Scheme 1). These hybrid structures (Figure 4D–F) will yield nanotubular silica after the LPEI core is removed by calcination.

**Figure 6:**
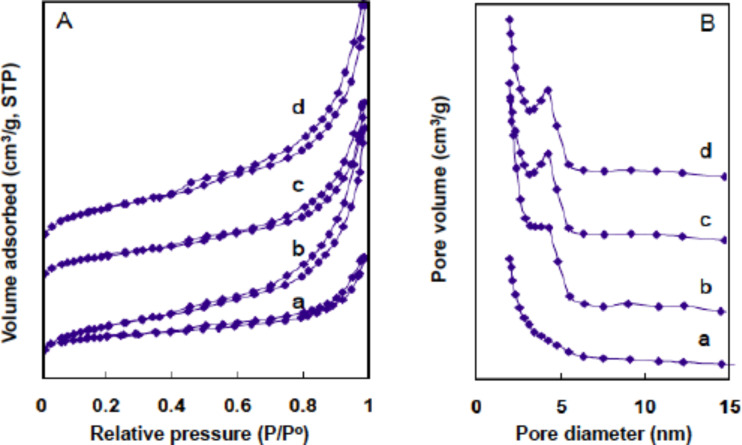
N_2_ adsorption/desorption isotherms (A) and BJH pore-size distribution curves obtained from the adsorption branch (B) of silica nanostructures synthesized by using silica source (MS51) concentrations of 23.1 wt % (a), 9.1 wt % (b), 4.6 wt % (c) and 1.1 wt % (d). LPEI aggregates was prepared by first dropping 1.0 mL of aqueous NaOH solution (5.0 M) into a mixture of 0.5 g of LPEI·HCl and 5 mL of water ([OH]/[EI] = 0.8), then washing the crystalline LPEI aggregates to pH 7.0 with centrifugation–redispersion cycles. The silica deposition conditions are the same as described in Figure 1.

**Effect of the silica source concentration.** We further found that the silica nanostructure could be controlled by changing the concentrations of the silica source. The LPEI fibrils prepared by using the molar ratio [OH]/[EI] = 0.8 with the same conditions as that used in Figure 1A–C were used in the silica deposition with different MS51 concentrations of 23.1 wt %, 9.1 wt %, 4.6 wt % and 1.1 wt %. SEM observation of the resulted products demonstrated that all the samples have a mat-like morphology (Figure S3 in Supporting Information File 1), which is similar to the silica nanotubes shown in Figure 1A–C. The N_2_ adsorption/desorption measurements provided important information about the difference in the nanostructures synthesized from the different concentrations of MS51. As shown in Figure 6, the silica structures that were synthesized from relatively low MS51 concentrations (9.1 wt %, 4.6 wt % and 1.1 wt %) exhibited BET surface areas from around 290 to 420 m^2^/g (runs 7, 8 and 9 in Table 1) with obvious type-IV hysteresis loops (Figure 6A), and peak values of around 4.2–4.3 nm from BJH pore-distribution curves (Figure 6B). This BET study confirmed a silica nanotube formation, which is consistent with that shown in Figure 1A–C. A remarkably different result is that the silica synthesized at high MS51 concentration of 23.1 wt % showed a significantly decreased BET surface area (244 m^2^/g, run 6 in Table 1) and a relatively smaller hysteresis loop (Figure 6A) without a peak value in the pore-size distribution (Figure 6B). This indicates the formation of silica as solid nanowires but not nanotubes. Presumably, a too high concentration of the silica source leads to an extremely high rate of hydrolysis and polycondensation of the alkoxysilane around the delicate LPEI fibrils, which could completely damage the crystalline LPEI. The collapsed LPEI then allowed for the silicification reaction to proceed continuously into the core domain of the hybrid nanostructures. Probably, the increased amount of the byproduct MeOH, which can dissolve LPEI aggregates, from this fast hydrolysis of high-concentration of oligomer methoxysilane also accelerates the collapse of the LPEI crystallites.

**Figure 7:**
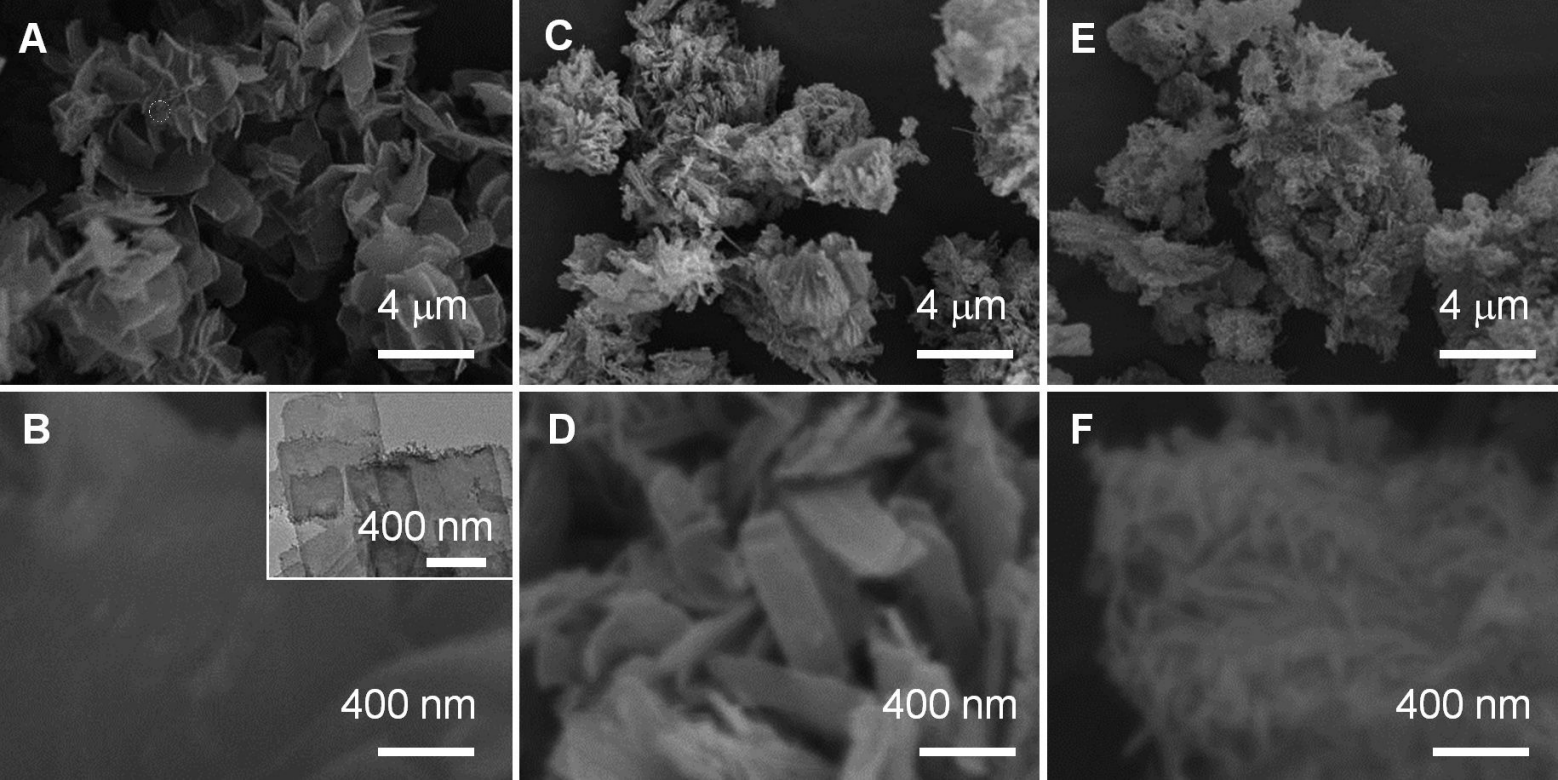
SEM images of silica structures, which were formed by adding different amounts of ammonia solution (NH_4_OH) to induce the crystallization-driven self-assembly of LPEI·HCl in water. The ratios of [OH]/[EI] for LPEI self-assembly are 0.6 (A and B), 0.8 (C and D) and 3.2 (E and F). The inset of B is a TEM image of the silica nanofilms. The silica deposition conditions are the same for A, B and C, which was performed by adding 1.5 mL of MS51 into 15 mL of aqueous dispersion of LPEI aggregates (pH 7.0) and stirring for 1 h at room temperature.

**Effect of the alkali basicity.** We also tried to use different alkalies to induce the room-temperature self-assembly of LPEI from the protonated state. The synthesis conditions are similar to that used for the silica formation induced by NaOH. Figure 7 shows the SEM images of silicas prepared by using ammonia solution (NH_4_OH) to induce self-assembly of LPEI with molar ratios [OH]/[EI] = 0.6, 0.8 and 3.2. We found that silica nanofilms were formed by using a molar ratio [OH]/[EI] = 0.6 (Figure 7A and Figure 7B), because the LPEI crystallization was delayed due to weak basicity of the ammonia solution and a low degree of deprotonation of LPEI–H^+^. This is consistent with the formation of silica nanofilms by using NaOH with low molar ratios [OH]/[EI]. Furthermore, when increasing the molar ratio [OH]/[EI] to 0.8, the templated silicification produced large silica aggregates, which were composed of ribbon-like structures (Figure 7C and D). As shown in Figure S4 (Supporting Information File 1), the BET measurement demonstrated that the N_2_ adsorption/desorption isotherm of this silica structure (see run 10 in Table 1) is similar to that synthesized by temperature-induced LPEI self-assembly (Figure 2). No peak value of the pore size distribution was observed (Figure S4 in Supporting Information File 1). To further confirm the effect of ammonia solution on the formation of silica nanostructure, the LPEI self-assembly was performed by using a molar ratio [OH]/[EI] = 3.2. As shown in Figure 7E and Figure 7F, large aggregates composed of a mixture of small silica nanoribbons and nanofibers were formed. The BET study did not show any obvious proof for a uniform formation of nanotube structures (run 11 in Table 1, Figure S4 in Supporting Information File 1).

The formation of this silica nanoribbons from an ammonia-induced LPEI template is dramatically different to the generation of silica nanotubes by NaOH-induced LPEI self-assembly with the same molar ratio of [OH]/[EI]. This could be partially attributed to the relatively weak basicity of ammonia solution compared to NaOH, which might not allow for a sufficient deprotonation of LPEI–H^+^ to cause the necessary rapid generation of LPEI aggregates. Similar silica nanoribbons were obtained by the use of LPEI aggregates formed from the deprotonation of LPEI–H^+^ by using the organic alkali Et_4_NOH (Figure S5A–C). In contrast, the utilization of relatively strong alkalies (KOH and LiOH) for the LPEI self-assembly produced the silica nanotube structures (Figure S5D–I). However, it should be noted that well-defined thin films of silica nanotubes could be synthesized even by using ammonia under the comparative conditions with using NaOH [37]. This could be attributed to the extreme excess of ammonia relative to the trace of LPEI–H^+^ adsorbed on substrate, which enables a rapid deprotonation reaction of LPEI–H^+^ on the substrate.

**Figure 8:**
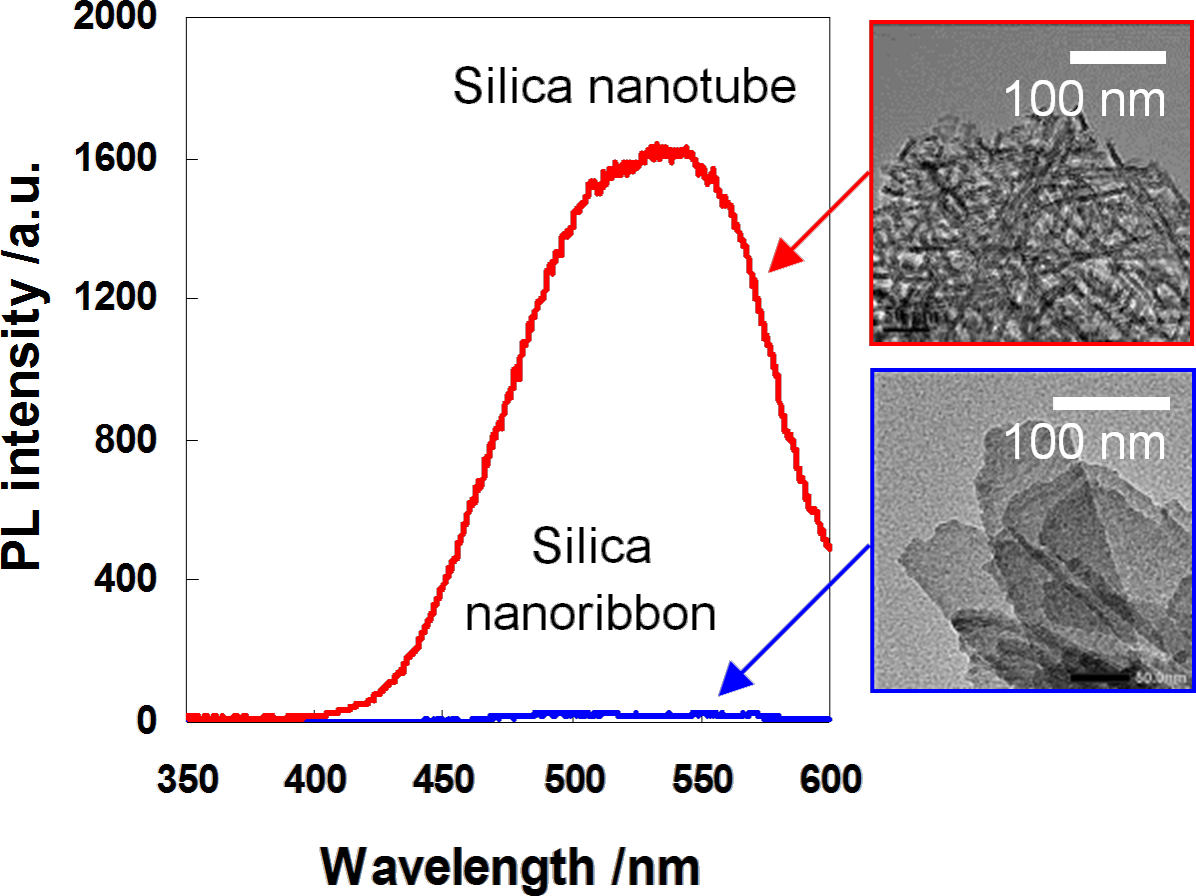
Phosphorescence emission spectra (λ_Ex_ = 320 nm) of silica nanotubes and nanoribbons that were synthesized by calcining the corresponding LPEI@silica hybrid nanotubes (Figure 1A–C) and nanoribbons (Figure 1D–F) under N_2_ atmosphere at 1000 °C.

**Photoluminescence.** It has been known that silica with a porous structure can be, after annealing, used as material, which emits visible light [41–44]. Although the reason of the silica photoluminescence is not clear, it is mainly attributed to the formation of oxygen defects on the SiO_2_ framework [44]. To examine the photoluminescence properties, the LPEI@silica hybrid nanostructures (nanotubes and nanoribbons, synthetic conditions given in Figure 1) were calcined at 1000 °C under N_2_ atmosphere. In general, the silica structures prepared from sol–gel procedures emit visible light with wavelengths smaller than 500 nm. However, as shown in Figure 8, a distinct emission centered at 540 nm was observed for the silica nanotubes under a maximum excitation wavelength of 320 nm. In comparison, no obvious emission was observed for the silica nanoribbons treated under the same N_2_-atmosphere pyrolysis conditions. In addition, the pure silica nanotubes calcined under air atmosphere did not show such emission (data not shown).

Although the detailed mechanisms of this special photoluminescent property of silica nanotubes are unknown at present, we expect two factors to be important in our case. One is the unique tubular structure with 3-nm wall and 3-nm inner hollow that would be in favor of generating geometrically stable photoactive sites, and the other one is the existence of trace carbon in the silica nanotubes (less than 0.9 wt % estimated by TGA) which would be doped into the silica frame to compose the photoactive sites. Probably, these two factors are vital for generating a green emission at over 540 nm. This is an interesting phenomenon and needs further investigation.

**Silica nanotube/carbon composite.** Silica/carbon composites are potentially applicable for electrochemical devices and selective solar absorber [45–46]. There have been some reports on the carbonization of porous silica–polymer [47] and organosilica/surfactant composites [48–49]. However, the synthesis of silica–carbon composite nanotube materials is still very rare. Liu and co-workers [50] have recently reported the fabrication of silica–carbon nanotubes by carbonization of organosilica nanotubes, which were synthesized by using expensive ethylene- or phenylene-bridged alkoxysilanes through the soft-template assembly method mediated by commercially available P123 in dilute solution.

**Figure 9:**
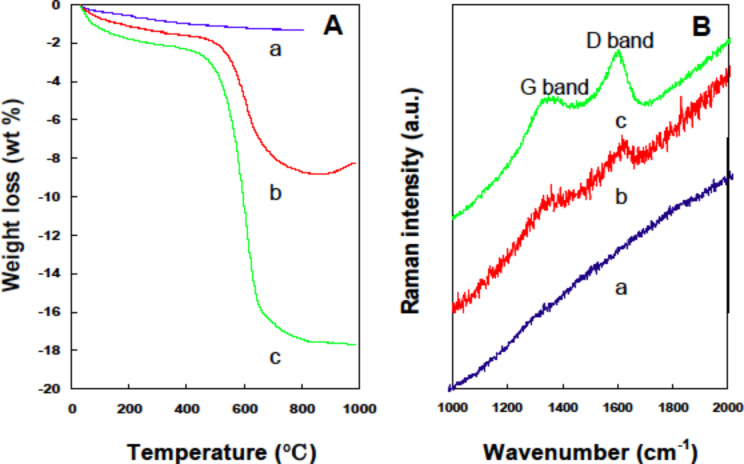
TGA (A) and Raman spectra (B) of silica/carbon composite nanotubes. The samples of a (0.8 wt % carbon), b (7.9 wt % carbon) and c (16.2 wt % carbon) were prepared by calcining LPEI@silica hybrid nanotubes (a), LPEI@silica nanotubes with one layer of adsorbed PSS (b) and two layers of adsorbed PSS (c) under N_2_ atmosphere, respectively.

**Figure 10:**
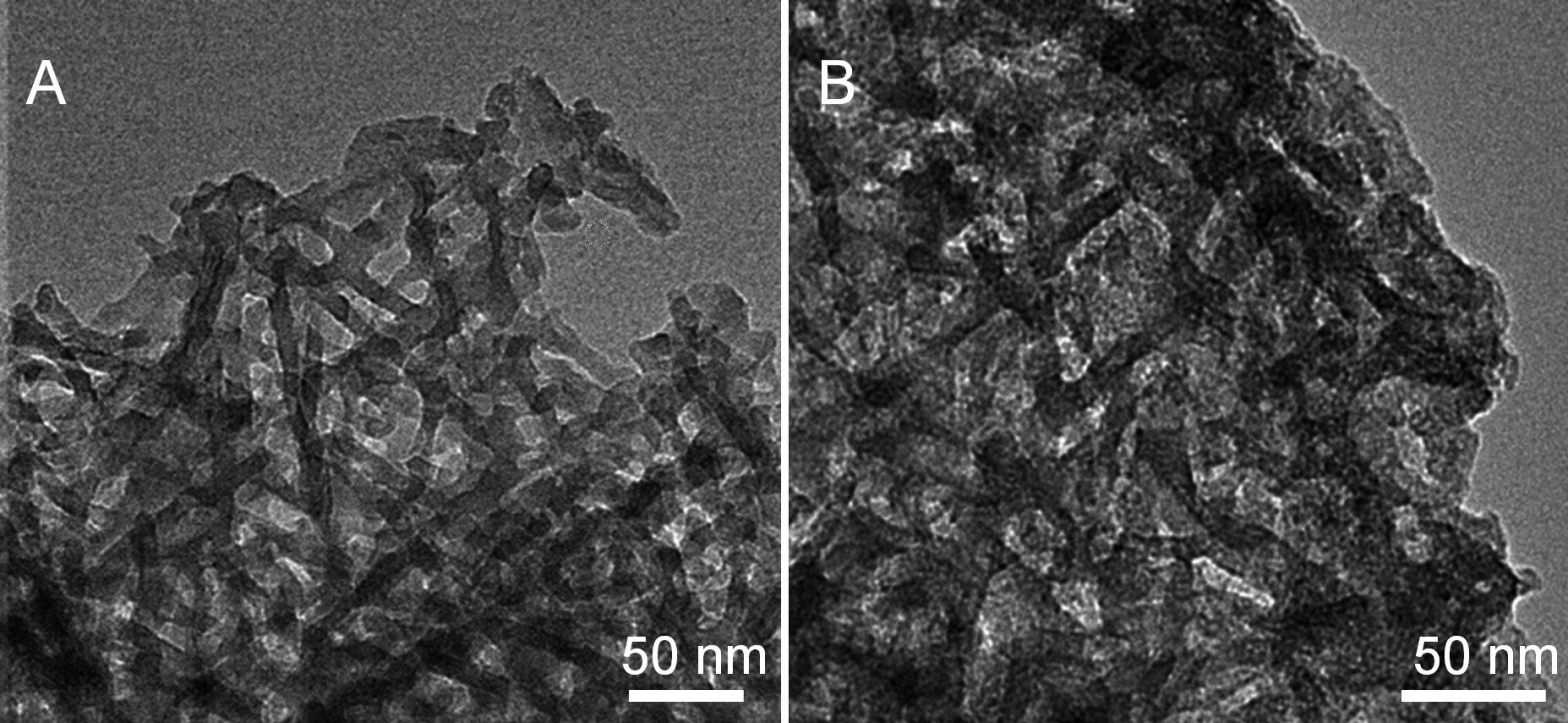
TEM images of silica/carbon composite mats with a nanofibrous network prepared by calcining LPEI@silica nanotubes under N_2_ atmosphere after PSS adsorption of one layer (A) and two layers (B).

We found that the direct carbonization of synthesized LPEI@silica hybrid nanotubes under N_2_ atmosphere (Figure 1A–C, LPEI content of 29 wt %) produced silica nanotubes with a carbon content of 0.84 wt % (“a” in Figure 9A). In order to increase the carbon content, we performed anionic adsorption of poly(4-styrenesulfonic acid) (PSS) PSS on the LPEI@silica nanotubes, which have a cationic surface because of the hybrid nature of the framework. The carbonization of the sample after the adsorption of one layer of PSS (polymer content: 47.9 wt %) produced silica/carbon composites with a much higher of carbon content (7.9 wt %, “b” in Figure 9A). The network structure of nanofibers of this silica/carbon composite was clearly observed by TEM observation (Figure 10A). The Raman spectrum shows very weak D and G bands at 1350 cm^−1^ and 1580 cm^−1^, respectively, which is indicative of the formation of disordered carbon in the composite (“b” in Figure 9B). We found that the carbon content in the composites could be further increased to 16.2 wt % (“c” in Figure 9A) by performing a second PSS adsorption (polymer content: 65.9 wt %). The Raman peaks of D and G bands ascribed to amorphous carbon then became stronger (“c” in Figure 9B). In the TEM images, one can still see a fibrous network in a carbon matrix, although the original thin mat of silica nanotubes became thicker (Figure 10B). These results reveal that silica/carbon hybrid nanotubes could be easily synthesized by making use of the hybrid nature of the LPEI@silica nanotubes and the composition of silica and carbon could be precisely controlled by polyelectrolyte adsorption.

## Conclusion

We have demonstrated that well-defined silica nanotubes of 10 nm diameter could be easily synthesized in solution through a templated silica mineralization on self-assembled LPEI fibrils, which in turn were formed by alkali-induced rapid deprotonation reaction of LPEI–H^+^ at room temperature. We demonstrated that a high molar ratio [OH]/[EI], a lower concentration of the silica source and stronger alkalies are important factors for the controlled formation of silica nanotubes. This approach is based on a widely-available low-cost synthetic polyamine and very mild silicification conditions (aqueous media, room-temperature and high efficiency), which could allow for an easy scale-up of silica nanotube fabrication and subsequently for wide technological applications. The silica nanotubes can be tuned to an emitter of visible light through the pyrolysis under N_2_ atmosphere. Moreover, silica/carbon composite nanotubes with tunable compositions could be synthesized by adsorption of a polyelectrolyte on LPEI@silica hybrid nanotubes and the subsequent carbonization.

## Experimental

**Materials.** LPEI·HCl with about 500 EI repeating units was synthesized by the hydrolysis of the precursor poly(ethyloxazoline) (*M*_w_ = 50,000, *M*_w_/*M*_n_ = 1.9, Aldrich) in an aqueous solution of 5 M HCl at 100 °C for 12 h, according to our previous method [35]. After hydrolysis, the white precipitates LPEI·HCl were washed with methanol for 3 times and dried in vacuum at 60 °C. LPEI was prepared by neutralizing LPEI·HCl in water, subsequently washing by water and acetone, and finally drying at 40 °C under vacuum. Poly(4-styrenesulfonic acid) (PSS, *M*_w_ = 75000, 18 wt % in water) was purchased from Aldrich. Methyl silicate 51 (MS51, 5-mer of tetramethoxysilane) was purchased from Matsumoto Chemical Co., Japan and was used as received. Other chemicals were used as received. Deionized water was used in all experiments.

**Synthesis of silica nanostructure by templating alkali-induced LPEI aggregates.** LPEI self-assembly were simply induced by dropping alkali (NaOH, NH_4_OH, KOH, LiOH or Et_4_NOH) into aqueous solution of LPEI·HCl with various molar ratios of [OH]/[EI] at room temperature. The solid fraction of LPEI aggregates appeared after the neutralized solution was washed with water and centrifuged until the pH of the supernatant reached a neutral value. The silicification was performed by adding MS51 into an aqueous dispersion of LPEI aggregates at room temperature. Specifically, a typical synthesis for silica nanotubes is as follows: 1 mL of an aqueous solution of NaOH (5 M) was dropped into a solution that contained 0.5 g of LPEI·HCl and 5 mL of water with a molar ratio [OH]/[EI] = 0.8. After the formation of self-assembled crystalline aggregates of LPEI, the mixture was washed with water for three cycles of centrifugation–redispersion. To 15 mL of an aqueous dispersion of LPEI aggregates (pH 7.2) was added 1.5 mL of MS51 and the mixture was stirred at room temperature for typically 1 h for the formation of LPEI@silica hybrid nanotubes. After water washing and drying, a white powder was obtained. LPEI@silica hybrid nanotubes were calcined by heating the sample up to 800 °C at a heating rate of 2.5 °C/min, and maintaining this temperature for 4 h under air atmospheres in order to completely remove LPEI and hence obtain pure silica nanotube.

**Synthesis of silica nanotube@carbon composite.** To increase the carbon content of silica/carbon composite nanotubes, an alternative adsorption of PSS and branched PEI was performed. 0.5 g of LPEI@silica hybrid nanotubes was dispersed into 30 mL of an aqueous solution of PSS (1 wt %), and the mixture was stirred at room temperature for 3 h for the adsorption of PSS. After centrifugation and washing with water, LPEI@silica@PSS (**S**) was obtained. The adsorption of branched PEI on **S** (**SE**) was conducted by dispersing **S** into 30 mL of aqueous solution of branched PEI (1 wt %) for 3 h. After a similar adsorption of PSS, LPEI@silica nanotubes with two PSS layers (**SES**) were finally obtained. The carbonization was performed by heating the samples firstly from room temperature to 900 °C at a heating rate of 2 °C/min and keeping the temperature constant at 900 °C for 8 h. Then the temperature was raised further up to 1000 °C at a rate of 2 °C/min and was held constant for 1 h.

**Characterizations.** Thermogravimetry analysis was performed on a TG-DTA 6300 instrument (SII Nano technology Inco., Japan). The measurement was conducted by heating LPEI@silica hybrid nanotube powders from 20 °C to 800 °C at a heating rate of 20 °C/min under air atmosphere. The morphology and nanostructure of silica was visualized by using a scanning electron microscope (SEM, Keyence, VE9800, Japan, working at 8 kV). The samples were sputter-coated with a thin layer of Pt prior to observation. Transmission electron microscopy (TEM) studies were conducted on a JEOL JEM-2200FS instrument operating at 200 kV. X-ray diffraction measurements (XRD) were carried out with a Rigaku RINT-TTR II diffractometer (Rigaku Co., Japan), using Cu Kα radiation (λ = 1.54 Å). Nitrogen sorption-isotherm measurements were performed on a Tristar 3000 volumetric adsorption analyzer (Micromeritics). Before the adsorption measurements, the samples were outgassed at 300 °C overnight. The degree of polycondensation (Q4, Q3 and Q2) of the LPEI@silica hybrid nanotube powders were characterized by ^29^Si CP-MAS NMR spectroscopy, and the spectra were recorded on a JEOL-400 MHz NMR spectrometer. Raman spectra were recorded with a Renishaw Raman imaging microscope. Radiation of 514 nm from an Ar-ion laser was used as the excitation source. The photoluminescence (phosphorescence) spectra of silica nanostructure were obtained with Hitachi F-4500 fluorescence spectrophotometer, in which the fluorescence emission was cut off electrically.

## Supporting Information

Supporting Information features SEM images, charts of N2 adsorption/desorption isotherms and pore size distributions of silica nanotubes synthesized under different conditions. In addition, a TGA profile of the hybrids that consisted of organic and silica nanotubes is given.

File 1 Additional SEM pictures, charts of N2 adsorption/desorption isotherms and pore size distributions, and TGA charts.
