# Supplementary material for: Controlled synthesis and tunable properties of ultrathin silica nanotubes through spontaneous polycondensation on polyamine fibrils
Source: Beilstein J Nanotechnol. 2013 Nov 25;4:793–804. doi: 10.3762/bjnano.4.90 (PMC3869340; doi:10.3762/bjnano.4.90)
Supplement: File 1 — Additional SEM pictures, charts of N2 adsorption/desorption isotherms and pore size distributions, and TGA charts. [file Beilstein_J_Nanotechnol-04-793-s001.pdf]

Supporting Information

for

Controlled synthesis and transformable properties of ultrathin silica nanotubes through spontaneous polycondensation on polyamine fibrils

Jian-Jun Yuan[1], Pei-Xin Zhu[1], Daisuke Noda[1] and Ren-Hua Jin*[2]

Address: [1]Synthetic Chemistry Lab., Kawamura Institute of Chemical Research, 631 Sakado, Sakura, 285-0078, Japan and [2]Department of Material and Life Chemistry, Kanagawa University, and JST-CREST 3-27-1 Rokkakubashi, Kanagawa-ku, Yokohama 221-8686, Japan

Email: Ren-Hua Jin - rhjin@kanagawa-u.ac.jp

*Corresponding author

Additional SEM pictures, charts of N2 adsorption/desorption isotherms and pore size distributions, and a TGA chart

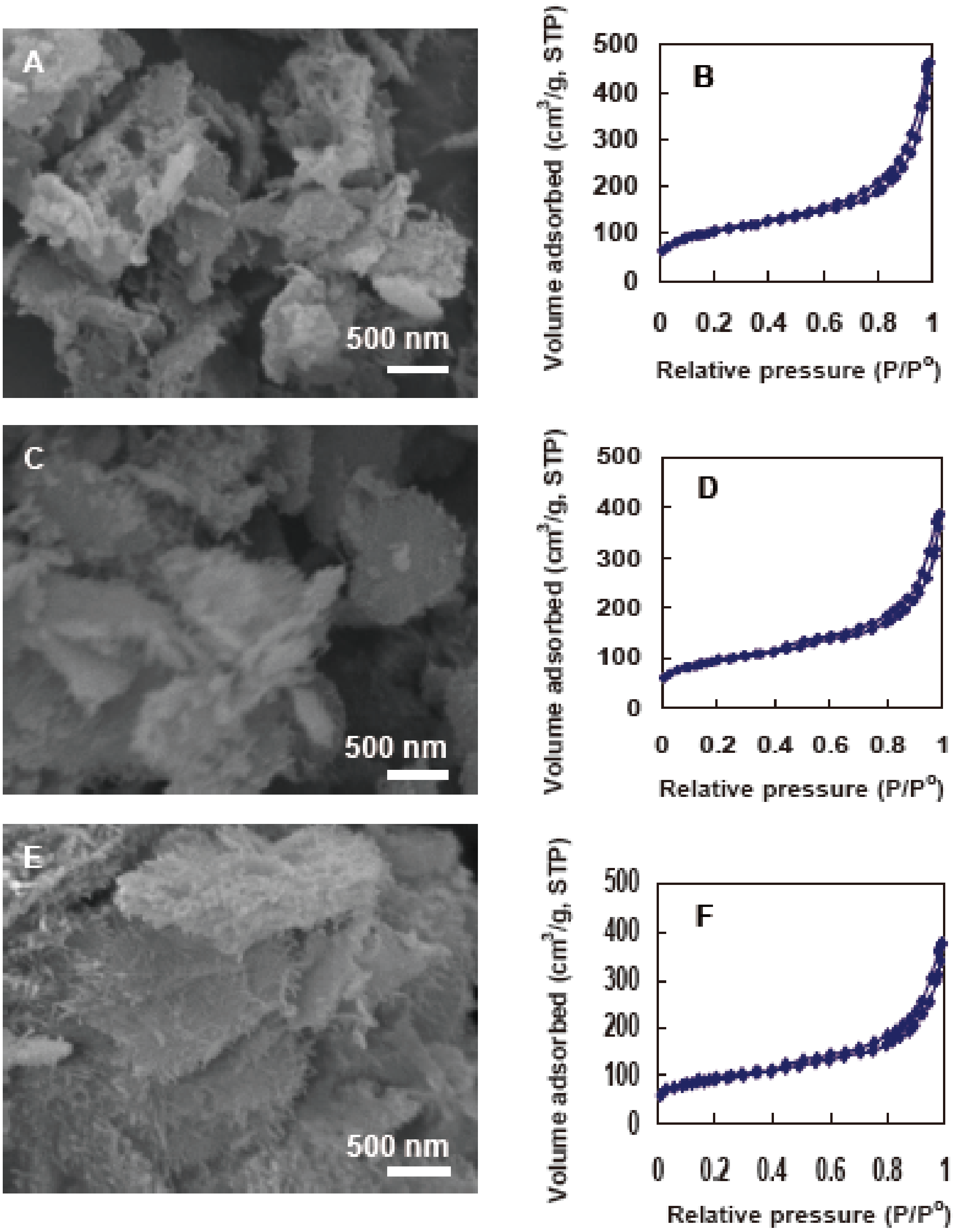

Figure S1: SEM images (A, C and E) and N_2 adsorption/desorption isotherms (B, D and F) of silica nanotubes synthesized with silicification times of 5 min (A and B), 60 min (C and D) and 240 min (E and F). The synthetic conditions for LPEI aggregates and silica deposition are the same as those used for Figure 3.

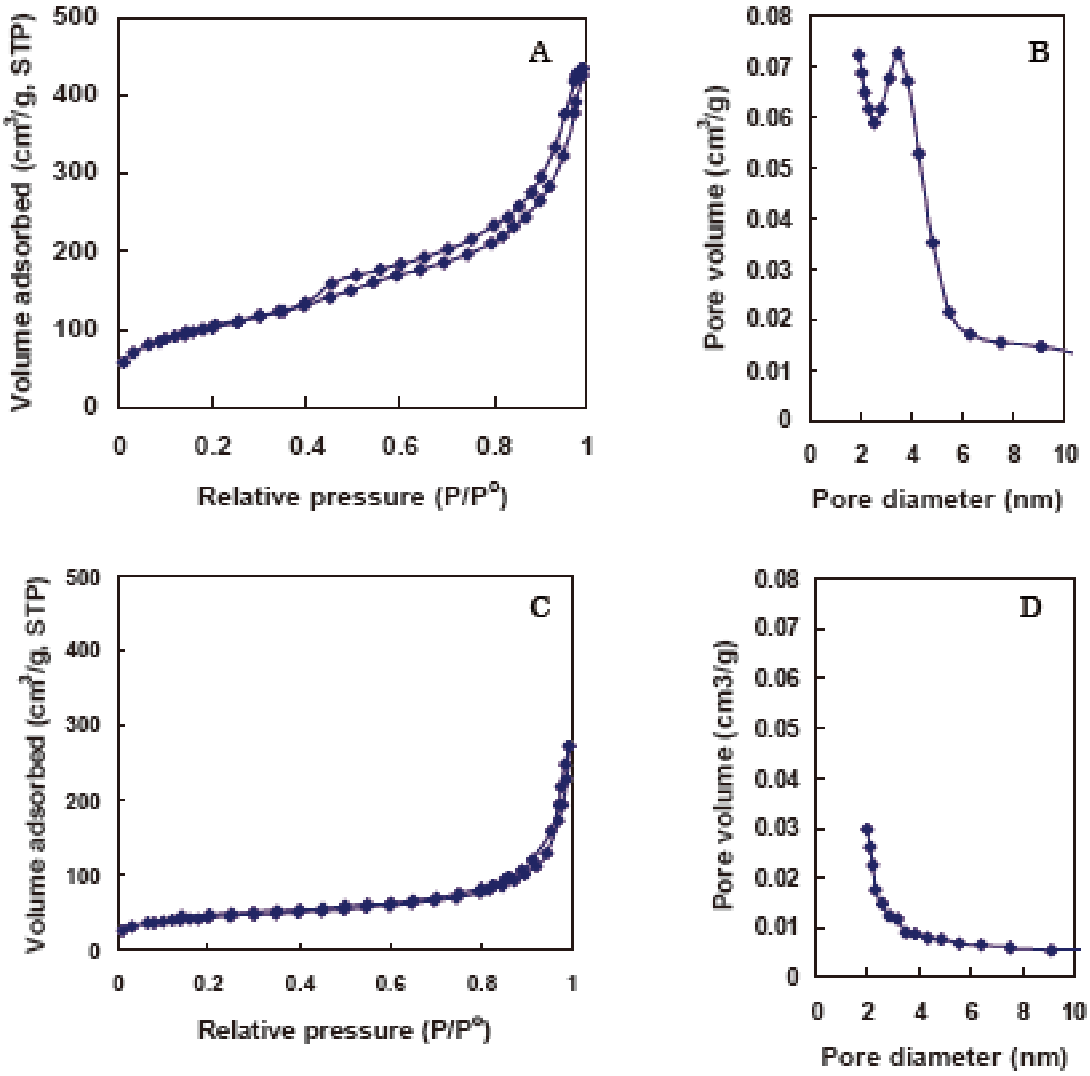

Figure S2: N_2 adsorption/desorption isotherms (A and C) and BJH pore-size distribution curve obtained from the adsorption branch (B and D) of silica nanotube (A and B) and silica nanoribbon (C and D). The synthesis conditions are the same as those of the samples shown in Figure 4. The sample was calcined at 800°C in air for 3h with at heating rate of 2.5°C per min.

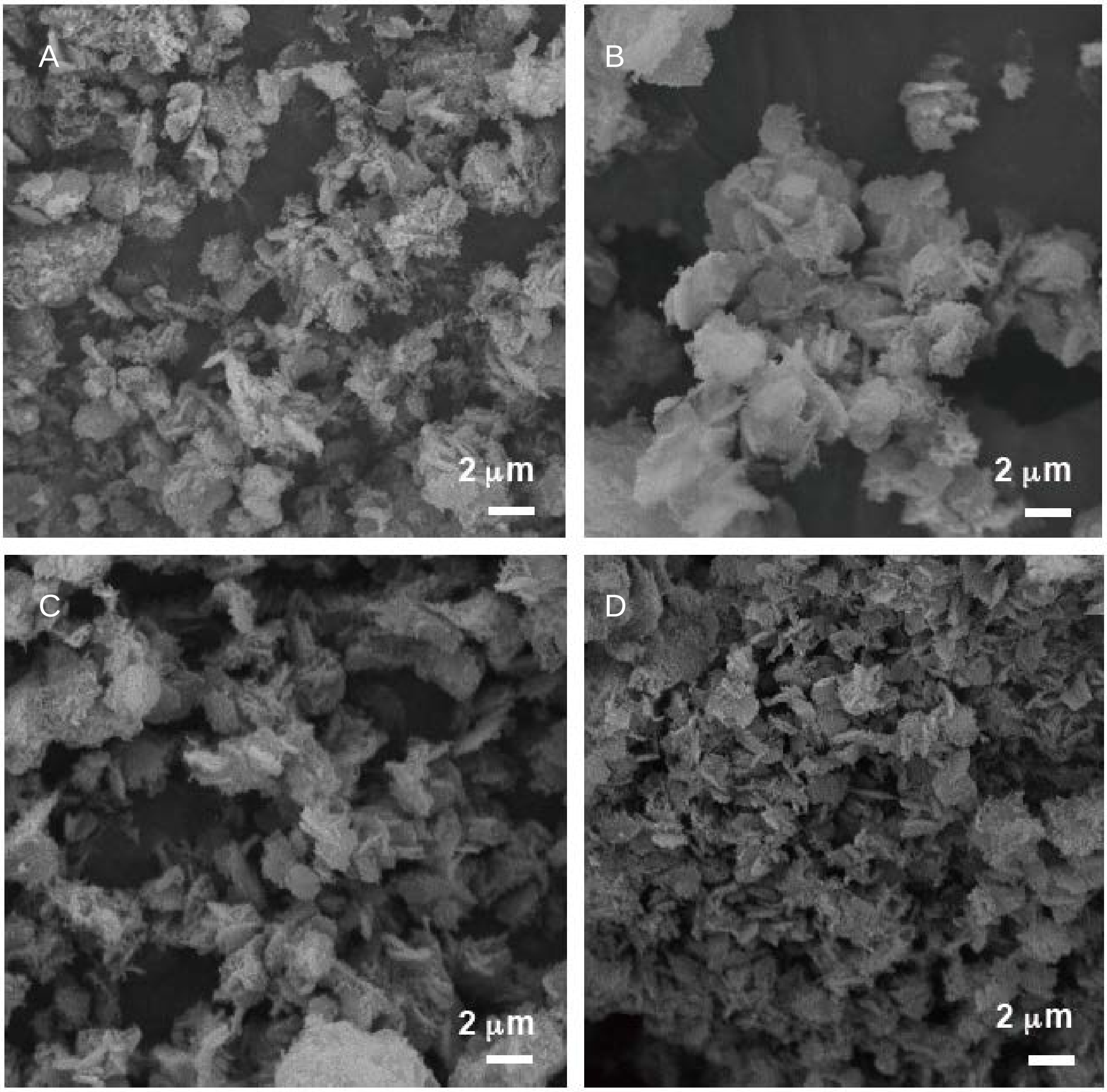

Figure S3: SEM images of silica nanostructure synthesized by using MS51 concentrations of 23.1 % (A), 9.1 % (B), 4.6 % (C) and 1.1 % (D). Other synthetic conditions are the same as those used in Figure 6.

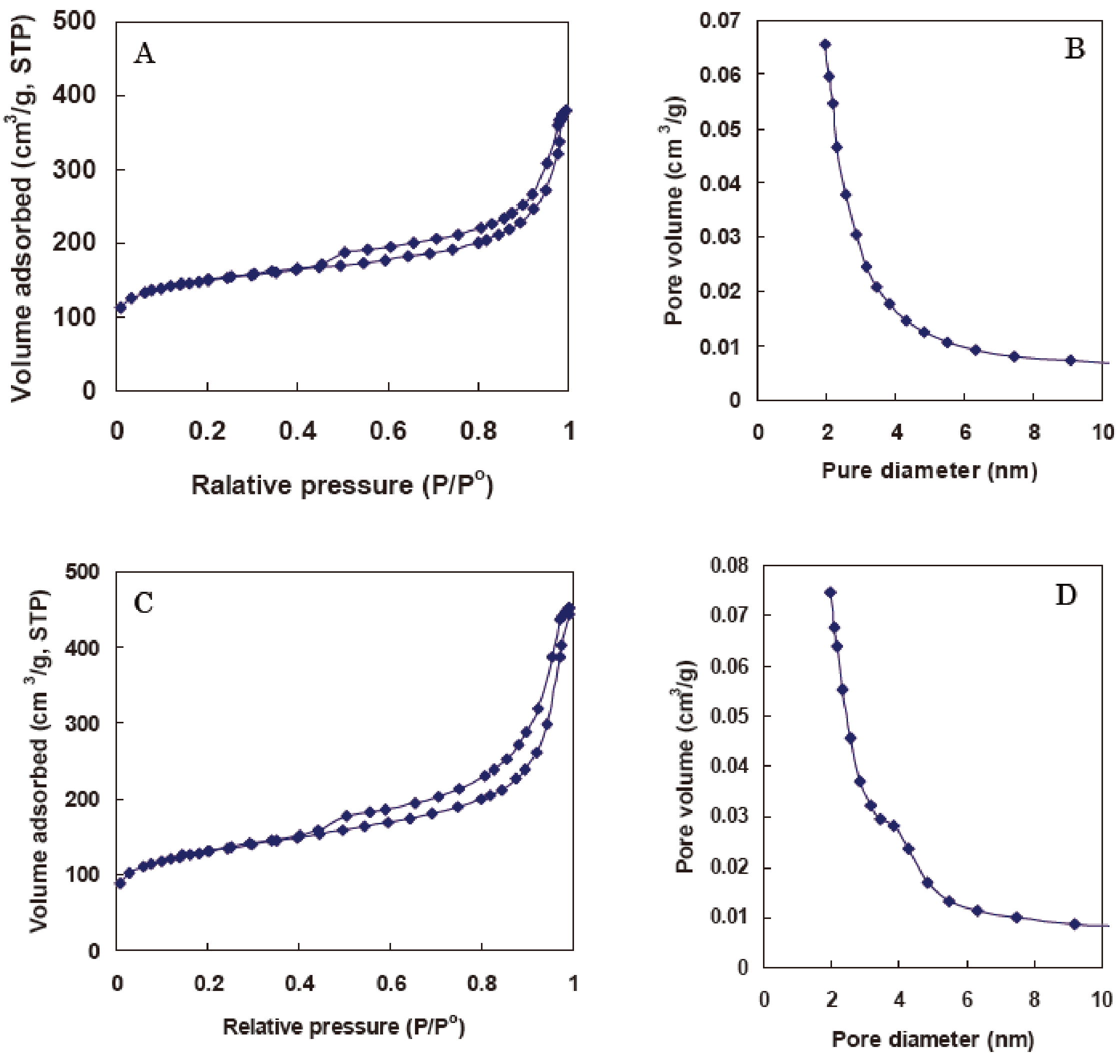

Figure S4: N_2 adsorption/desorption isotherms (A and C) and BJH pore-size distribution curve obtained from the adsorption branch (B and D) of nanostructured silicas synthesized under the feeding ratios of [OH]/[EI] of 0.8 (A and B) and 3.2 (C and D). The synthesis conditions are the same as those of the samples shown in Figure 7. The sample was calcined at 800°C in air for 3h with at heating rate of 2.5^{o}C per min.

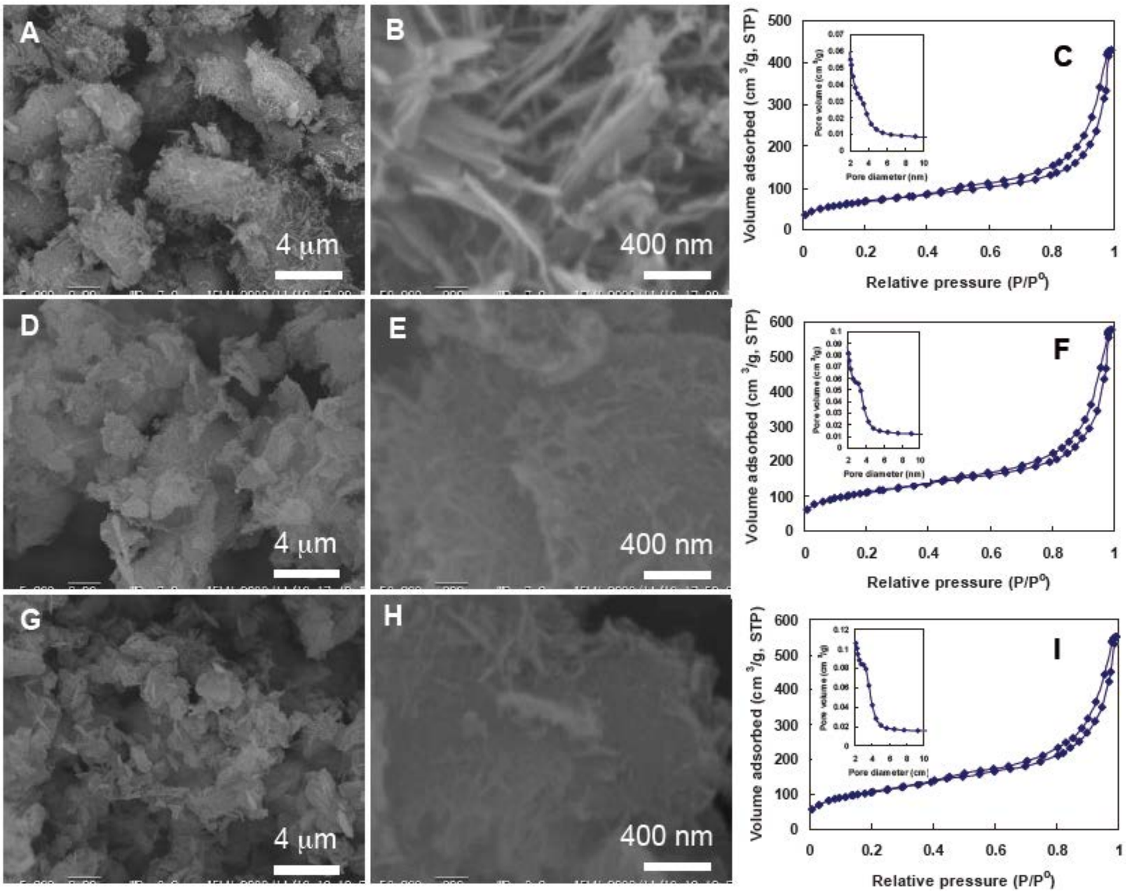

Figure S5: SEM images (A, B, E, F, H and I) and BET results (C, G and J) of nanostructured silicas synthesized by using bases of Et_4NOH ([OH]/[EI] = 1.6) (A–C), LiOH ([OH]/[EI] = 2.7) (D–F) and KOH ([OH]/[EI] = 3.2) (H–J). 0.5 g of LPEI-HCl was dissolved into 6 mL of water for LPEI self-assembly induced with various bases. The silica deposition was performed by stirring a mixture of 1.5 mL of MS51 and 15 mL of aqueous dispersion of LPEI aggregates for 4 h at room temperature.

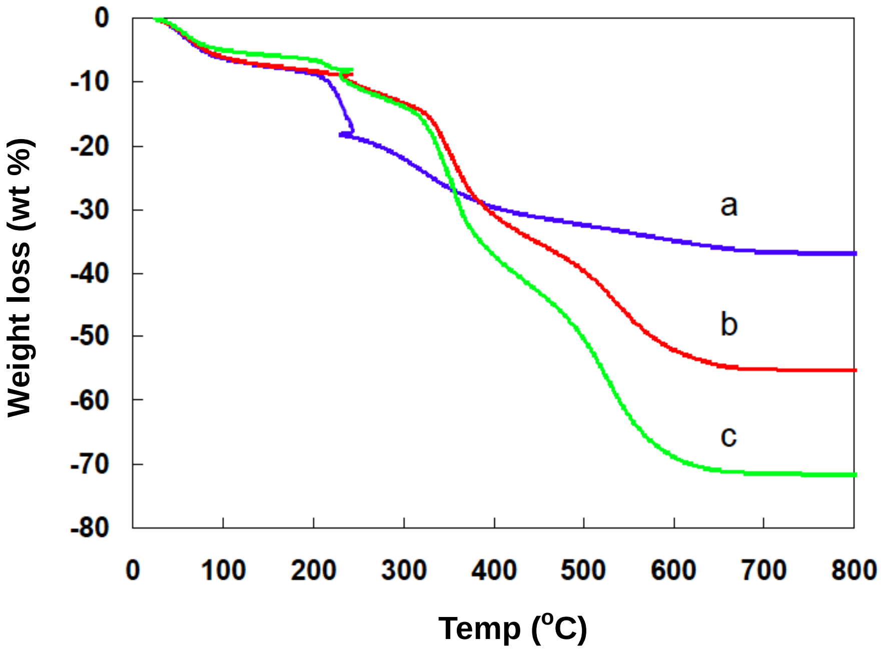

Figure S6: TGA curves of LPEI@silica hybrid nanotubes (a), and the nanotubes adsorbed with one layer of PSS (b) and two layers of PSS (c).
